# Healing by Secondary Intention or Skin Grafting for the Paramedian Forehead Flap Donor Area

**DOI:** 10.7759/cureus.77076

**Published:** 2025-01-07

**Authors:** Diogo Conduto, Afonso Antunes de Almeida, Andre Pinto, Artur Nixon Martins, Carlos Pinheiro

**Affiliations:** 1 Plastic Surgery, Hospital de Santa Maria - ULSSM (Unidade Local de Saúde Santa Maria) - Lisboa, Lisbon, PRT; 2 Institute of Anatomy, Faculdade de Medicina da Universidade de Lisboa, Lisbon, PRT

**Keywords:** donor site management, nasal reconstruction, paramedian frontal flap, secondary intention healing, skin grafting

## Abstract

Nasal reconstruction aims to restore both function and aesthetic integrity, with the nose being central to facial harmony. The paramedian forehead flap (PFF) is a cornerstone of nasal reconstruction due to its reliable vascular supply and excellent tissue match. Managing the PFF donor area, especially for larger defects, presents challenges, with secondary intention healing and skin grafting being common approaches.

A 72-year-old male underwent nasal reconstruction with a PFF after basal cell carcinoma excision. The flap's donor area was managed with a skin graft. Years later, the patient developed a nasal squamous cell carcinoma requiring partial rhinectomy, followed by staged nasal reconstruction. The process included another PFF, providing an opportunity to compare two donor area management techniques: skin grafting and secondary intention healing. This case highlights the outcomes of both approaches, supported by a detailed photographic report documenting their differences in terms of aesthetics and patient satisfaction.

This case provides insights into the optimal management of the PFF donor area. It allows a direct comparison between secondary intention healing and skin grafting, which are the conventional options, highlighting distinct outcomes in terms of aesthetics and patient satisfaction.

## Introduction

Nasal reconstruction aims to restore both function and aesthetics, with the nose serving as the facial centerpiece. The paramedian forehead flap (PFF) is widely regarded as the workhorse reconstructive option for nasal superficial coverage due to its excellent color and texture match, as well as its reliable vascular supply from the supratrochlear artery, making it suitable for both simple and complex cases [1,2].

Managing the flap donor area, however, is not always straightforward. While smaller defects (<2.5-3.5 cm in width) are typically closed primarily, larger defects may require healing by secondary intention or coverage with a skin graft [3,4].

Healing by secondary intention can achieve good color matching with native skin but carries risks such as bone exposure, prolonged wound care, and the potential need for reintervention [5]. Skin grafting, on the other hand, can provide comparable texture and thickness but may result in color mismatch, graft failure, and increased morbidity, potentially compromising aesthetic outcomes [6].

This case report aims to compare secondary intention healing and skin grafting for managing medium to large PFF donor areas. By presenting a patient where both strategies were employed at different times by different teams, we offer a direct comparison of their outcomes, contributing valuable insights to this ongoing discussion.

## Case presentation

**Figure 1:**
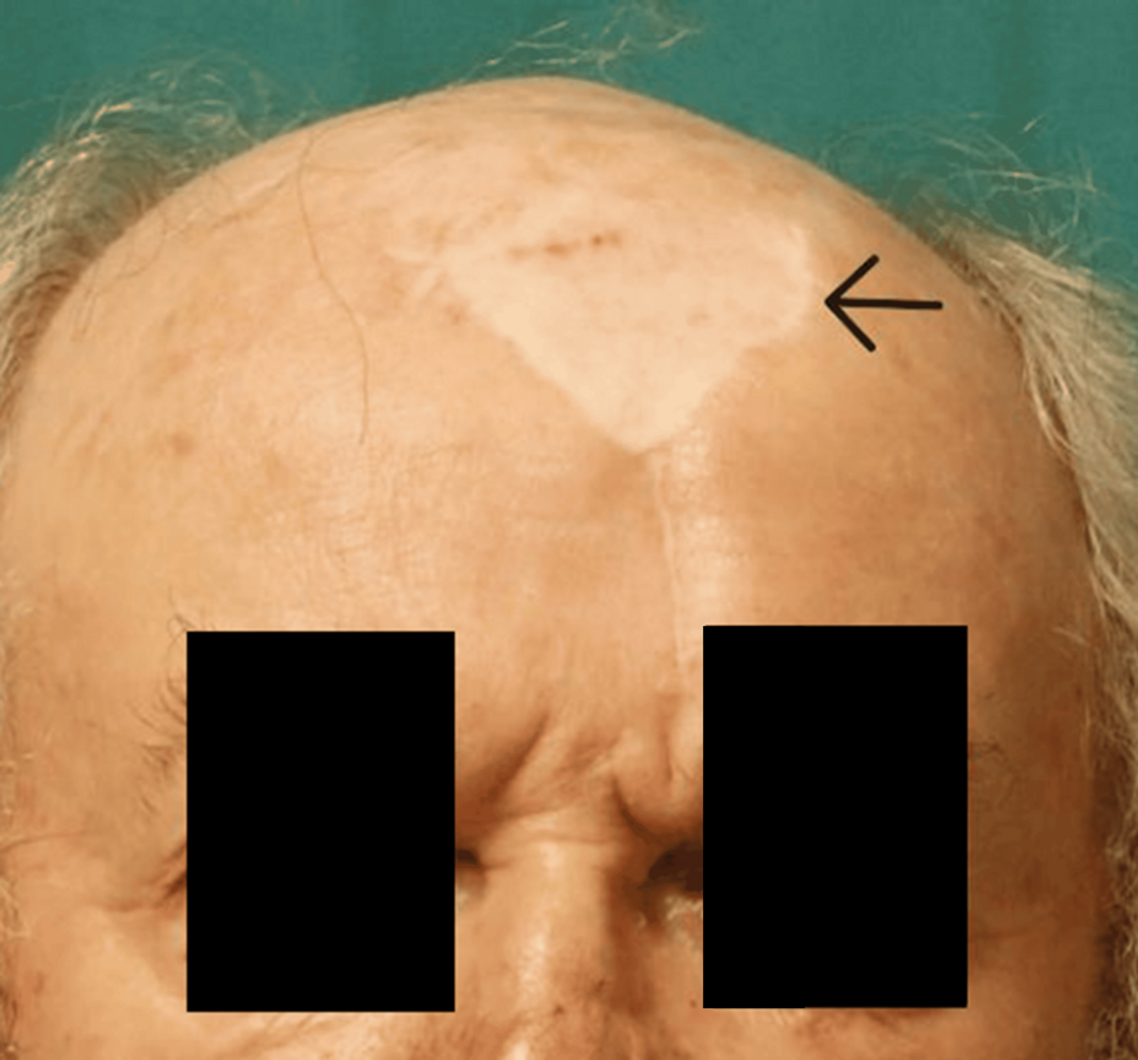
Seven-year follow-up after the first nasal reconstruction using a paramedian forehead flap. The donor area, closed with a skin graft, is indicated by a black arrow.

A 72-year-old male patient presented with a cutaneous lesion on the right nasal wing. A biopsy was performed, and the histopathological report confirmed a micronodular infiltrative ulcerated basal cell carcinoma with areas of fibrosis, invading up to the muscular planes at the inferior margin of the excision. Mohs surgery was conducted, resulting in a significant nasal defect involving both cutaneous and cartilaginous components. The defect was reconstructed using a mucoperichondrial nasal septum flap, an auricular conchal graft, and a left paramedian forehead flap (PFF). The secondary forehead defect measured approximately 6 cm in width and was closed with a full-thickness skin graft harvested from the supraclavicular region. The post-operative period was uneventful, and the outcome after 7 years is shown in Figure 1.

Seven years later, the patient presented with a new nodule in the area of the previous nasal reconstruction flap, accompanied by episodes of spontaneous epistaxis. Clinical examination identified infiltration on the internal surface of the right nasal choana, with suspected involvement of the nasal septum.

A biopsy confirmed invasive, moderately differentiated squamous cell carcinoma, partially keratinizing. A contrast-enhanced CT scan revealed asymmetry of the nasal structure, particularly at the right alar margin, with thickening of the overlying skin and subcutaneous tissue. This extended laterally to the right nasolabial sulcus. The lesion measured 15 mm in maximum thickness and involved the anterior aspect of the right nasal cavity, with contact with the nasal septum. There was no evidence of erosion of the maxillary or ethmoidal bones. Additional findings included multiple small cervical lymph nodes, none of which met the criteria for adenomegaly.

Given the extent of the malignancy, a partial rhinectomy was performed as the oncological treatment. Reconstruction followed the principles described by Frederick J. Menick [1]. A left radial forearm free flap was used to restore the nasal lining and cutaneous surface. A subsequent procedure involved introducing a cartilage graft for structural support, with the nasal surface reconstructed using a right PFF. Unlike the previous surgery, the distal donor site of the PFF, measuring approximately 3.5 cm in width, was left to heal by secondary intention. Dressings included petrolatum-impregnated gauze covered with iodine-impregnated gauze. The postoperative course was uneventful.

**Figure 2:**
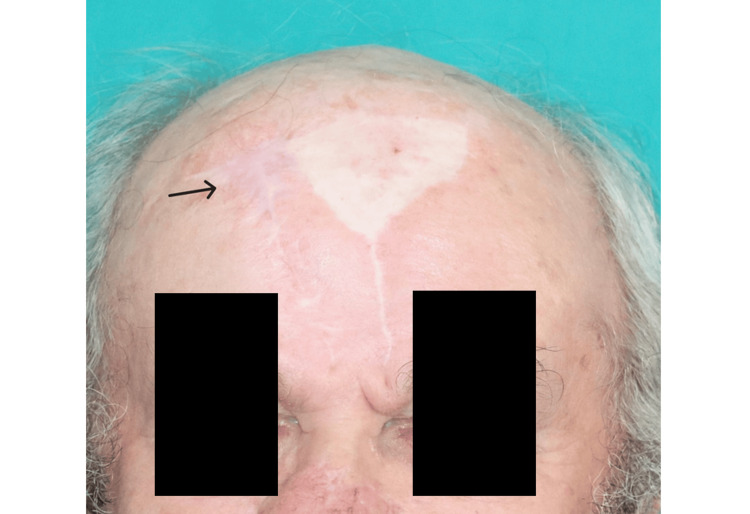
One-year follow-up after the reconstruction of a nasal defect with a second paramedian forehead flap. The donor area, left to heal by secondary intention, is indicated by a black arrow.

At the one-year follow-up, the patient reported greater satisfaction with the aesthetic appearance of the forehead scar left to heal by secondary intention although he noted increased pruritus compared to the skin-grafted donor site. Both PFF donor areas are shown in Figure 2.

## Discussion

Managing the donor area of the paramedian forehead flap (PFF) requires careful consideration. For flaps with a narrow base (approximately 1.5 cm), the caudal portion is typically closed primarily. Some smaller cephalic defects (<2.5 cm in width) may also be amenable to primary closure. However, for larger defects, surgeons must choose between healing by secondary intention or skin grafting.

Secondary intention healing often involves extensive submuscular undermining of the forehead to achieve maximum tissue mobilization. This technique reduces tension during the closure of the caudal portion and minimizes the size of the cephalic defect, allowing it to heal more effectively by secondary intention. A critical aspect of this approach is maintaining a moist environment to prevent periosteal necrosis, as drying of the exposed periosteum can delay healing and necessitate secondary interventions [3]. Wound contraction plays a significant role in reducing scar size while reepithelialization promotes a scar that blends in color and texture with the surrounding skin, enhancing the aesthetic outcome.

Skin grafting offers a predictable healing process characterized by imbibition, revascularization, and maturation. Typically, the defect is considered “reconstructed” within one week of graft application. Over time, the graft undergoes contraction and remodeling, a process that can take up to two years to complete [6]. While skin grafting offers quicker healing and a uniform graft appearance, it has notable disadvantages, including a higher likelihood of color and texture mismatch with the recipient area, reduced sensation, and risks of early complications such as infection or hematoma, which may compromise graft viability.

When comparing the two methods, secondary intention healing offers superior integration of color and texture, resulting in a scar that blends naturally with the forehead skin over time. However, it requires a longer healing period and may initially result in more noticeable scarring. Studies indicate that secondary intention healing promotes significant wound contraction, leading to a markedly reduced wound size by the end of the maturation process [7]. This phenomenon is attributed to the absence of dermis in the wound area, as dermis inhibits contraction [8-10]. In contrast, skin grafting provides a faster solution but often at the expense of aesthetic outcomes and additional morbidity at the donor site. While it offers the advantage of quicker initial healing, its long-term integration may be less harmonious than secondary intention healing.

The presented case provides a unique opportunity to directly compare both techniques for managing the PFF donor area. This comparison is particularly rare, as the need for two PFFs in the same patient is uncommon and even more so when different approaches are employed for the donor area. This scenario was made possible because the patient’s initial treatment for the first tumor occurred at a different hospital, where a distinct surgical philosophy was applied.

In the end, both the patient and the surgical team concluded that healing by secondary intention resulted in a more natural and aesthetically pleasing outcome. As illustrated in Figure 2, secondary intention healing produced a scar that blended seamlessly with the surrounding facial contours, demonstrating superior color matching and reduced size. In contrast, the skin-grafted area appears more conspicuous, with starkly defined borders and a noticeable mismatch in color, drawing attention to the donor site. These differences, evident in the images, underscore the clinical significance of secondary intention healing in achieving optimal aesthetic results. Additionally, this approach eliminates the morbidity and scarring associated with harvesting a secondary donor site, further reinforcing its value for defects that cannot be closed primarily.

When considering cost-effectiveness, secondary intention healing offers notable advantages. Its superior aesthetic outcomes and the use of simple, inexpensive dressings make it an appealing option. However, this approach may require longer wound care, potentially increasing costs associated with transportation and qualified personnel for dressing changes. In cases of very large defects, particularly in patients where aesthetic benefits are less significant, the prolonged care requirements of secondary intention healing may outweigh its advantages, making skin grafting a more cost-effective alternative.

## Conclusions

The management of the PFF donor area depends largely on the defect’s size. This case highlights that healing by secondary intention provides superior aesthetic outcomes compared to skin grafting, despite requiring a longer recovery period, consistent with findings in the literature. An individualized approach, tailored to patient-specific factors and incorporating considerations of cost-effectiveness, is crucial for optimizing outcomes in nasal reconstruction.
